# Clinical presentations and molecular characterization of Indonesian children with 5 alpha-reductase deficiency

**DOI:** 10.1186/1687-9856-2013-S1-P191

**Published:** 2013-10-03

**Authors:** Nanis S Marzuki, Lita P Suciati, Firman P Idris, Bambang Tridjaja

**Affiliations:** 1 Eijkman Institute for Molecular Biology, Jakarta, Indonesia; 2 Pediatric Endocrinology Division, Department of Child Health, Faculty of Medicine, University of Indonesia

## 

Inactivating mutations in 5 alpha-reductase (SRD5A2) gene lead to steroid 5 alpha-reductase deficiency, a rare autosomal recessive condition affecting 46,XY individuals, who generally present with clinical spectrum ranging from a male phenotype with hypospadia to a female phenotype with normal wolffian structures. More than 50 different mutations responsible for this disorder have been documented. This study is to extend our previous report, which described two siblings with 2 novel mutations of SRD5A2 gene, on Indonesian children with 5 alpha-reductase deficiency.

We describe clinical, hormonal, and molecular profiles of 14 children with 46,XY DSD and undervirilization due to 5-alpha reductase deficiency. DNAs extracted from peripheral blood lymphocytes, and the 5 exons of SRD5A2 gene were amplified using specific primers and sequenced.

All of the patients (aged 2 months-18 years old) presented with genitalia ambiguity, including variable degree of hypospadia, phallic enlargement, palpable testis in ‘girls’, micropenis, cryptorchidism, virilization during puberty in ‘girls’ (8 out of 12 patients), and dismorphic appearances in 1 case. Twelve of them raised as girls, while eight patients who had entered pubertal age changed their identity to male. The T/DHT ratio were more than 20 in 8 patients with documented hormonal results. Three mutations including p.Gly34Fs, c.699-1 G>T, and p.Glu135Lys, which have not been reported in other populations were detected. The p.Arg227Gln was the most frequent mutations found (in 13/14 patients or 13/28 alleles). The combination of p.Val89Leu and p.Arg227Gln, which rarely reported in other populations, were detected in 6 patients.

In our series, who all presented with genitalia ambiguity and mostly raised as girls, three mutations, including p.Gly34Fs, c.699-1 G>T, and p.Glu135Lys, which never been reported in other populations were detected.

